# Fra-1 regulation of Matrix Metallopeptidase-1 (MMP-1) in metastatic variants of MDA-MB-231 breast cancer cells

**DOI:** 10.12688/f1000research.2-229.v1

**Published:** 2013-10-29

**Authors:** Eric Henckels, Ron Prywes

**Affiliations:** 1 Department of Biological Sciences, Columbia University, New York, NY 10027, USA

## Abstract

Matrix Metallopeptidase 1 (MMP-1) expression has repeatedly been correlated to tumorigenesis and metastasis.  Yet, MMP-1 regulation in a metastatic context remains largely unknown.  Here we confirm differential MMP-1 expression in mammary carcinoma cells with varied metastatic potentials. We show that MMP-1 expression is regulated by an AP-1 element in its promoter in highly metastatic MDA-MB-231 mammary carcinoma cell derivatives.  Fra-1, an AP-1 family transcription factor, differentially binds this element in highly metastatic cells compared to low metastatic cells and is required for MMP-1 expression.  Overexpression of Fra-1 also caused increased MMP-1 expression. Fra-1 mRNA levels are unchanged in the cell variants, however its protein levels are higher in the metastatic cells. While there was no change in Fra-1 protein degradation rates, protein synthesis of Fra-1 was increased in the metastatic cell variant. These results demonstrate that Fra-1 and MMP-1 levels are differentially regulated in metastatic cell variants at the level of Fra-1 protein translation. Consistent with the importance of Fra-1 for tumor growth, we found that Fra-1 overexpression was sufficient to increase cell motility and anchorage independent growth.  These results suggest that increased Fra-1 translation is critical for regulation of MMP-1 and tumor cell metastasis.

## Introduction

Matrix metallopeptidase-1 (MMP-1) expression is highly correlated to several forms of cancer ^1^. In breast cancer patients, MMP-1 expression has been correlated to primary tumor progression, metastatic potential, and survival ^2– 6^. Further, in glioblastoma, melanoma and breast cancer, higher incidence has been associated with a single nucleotide polymorphism in an Ets-binding site which increases MMP-1 expression ^7, 8^.

Outside the clinic, MMP-1 expression has been measured in a variety of breast cancer cell lines. In general, its expression is greater in cells with higher metastatic potential (e.g. MDA-MB-231) when compared to cells of low metastatic potential (e.g. MCF-7) ^9– 11^. Similarly, MDA-MB-231 cell variants with different metastatic potentials demonstrate the correlation between MMP-1 expression and metastasis ^12– 14^.

MMP-1 regulation has been well studied in HeLa and other cell culture systems ^15^. However, less is known about how MMP-1 is regulated in metastasis. Recent studies have identified several promoter regions and factors that may play a role in MMP-1 regulation. For example, in melanoma cells, Twist binding to the MMP-1 promoter was found to increase expression of MMP-1 ^16^. In MCF-7 cells, HER2, which is upregulated in 15% – 25% of breast tumors and associated with poor prognosis, was found to upregulate MMP-1 through the ERK1/2 pathway ^17^.

The AP-1 consensus site is the archetype for tumor associated gene expression. It was discovered in the MMP-1 promoter as being activated by tumor promoting phorbol esters ^18, 19^. Since its discovery, the role of AP-1 in tumorigenesis has been further substantiated ^20^. Tissue immunohistochemistry revealed that expression of Fra-1, an AP-1 family member, correlates with breast cancer malignancy ^21, 22^.

To study the altered regulation of gene expression in metastatic breast cancer cells, we have utilized a series of MDA-MB-231 breast adenocarcinoma cell variants developed by the Massague lab ^12, 14^. MDA-MB-231 cells, which were derived from a pleural effusion of a breast cancer patient with relapsed disease, cause a low level of metastasis when injected into immunocompromised mice by various routes. Metastatic cells from these xenografts had greater metastatic potential when subsequently cultured and reinjected into mice. These cells derived from metastatic tumors in secondary organs (e.g. lung and bone) also showed greater organ-specificity. Alternatively, MDA-MB-231 cells were single cell cloned. These propagated single cell populations (Scp cell lines) had varied metastatic potential. Analysis of genomic expression using microarrays on these cell lines of varying metastatic potential provided us with an opportunity to identify genes correlated with metastatic potential ^12– 14^. In addition, these cell lines provided us with a well controlled system to understand the mechanism of how gene expression is altered in highly metastatic cells.

The gene whose expression was most strongly increased in the highly metastatic cell variants was MMP-1. In this study we have compared the expression of MMP-1 in the high and low metastatic MDA-MB-231 variants and present evidence for the role of an AP-1 site in the MMP-1 promoter and the translational regulation of the AP-1 family member Fra-1.

## Materials and methods

### Analysis of microarray data

Microarray gene expression data was available as supplemental data in several publications ^12– 14^ ( http://www.sciencedirect.com/science/MiamiMultiMediaURL/1-s2.0-S1535610803001326/1-s2.0-S1535610803001326-mmc1.xls/272618/FULL/S1535610803001326/8e4e6bf4cf8c1acc68ed4588b192b303/mmc1.xls, http://www.sciencedirect.com/science/MiamiMultiMediaURL/1-s2.0-S1535610803001326/1-s2.0-S1535610803001326-mmc2.xls/272618/FULL/S1535610803001326/2c83b94783f578fb7aeff9fb3c2b6c0d/mmc2.xls, http://www.nature.com/nature/journal/v436/n7050/extref/nature03799-s10.xls). To parse the data, Affymetrix comparison sheets were used with Microsoft Excel Vlookup functions to match primer coding with gene name, symbol and reference sequence ID. Expression values from cell lines with high metastatic potential to the bone (1833, Scp-2, Scp-25 and Scp-46), to the lung (1834, 3481, 4142, 4173, 4175, 4180, Scp-3 and Scp-28), or with low metastatic potential (MDA-MB-231, Scp-6, Scp-21 and Scp-26) were averaged for each gene. The ratio of high to low metastatic potential expression levels was calculated and ordered by highest ratio. A T-test (two tailed distribution, equal variance) using Microsoft Excel was used to calculate the p value for the significance of the differences between each group.

### Cell culture

Scp-2, Scp-3, Scp-21, Scp-26, Scp-28, and MDA-MB-231 cell lines were a generous gift from Joan Massague (Memorial Sloan Kettering Research Institute) ^14^. Cells were grown in Dulbecco’s modified Eagle’s media (DMEM) supplemented with 10% fetal bovine serum (Gemini Bio-Products). Phoenix amphotropic helper cells from Gary Nolan (Stanford University) ^23^ were grown in DMEM supplemented with 10% Fetal Bovine Serum.

Cell lines stably expressing Fra-1 or a control vector were made in Scp-21 cells. The Fra-1 retroviral expression vector, p6599 MSCV-IP N-HAonly FosL1 ^24^, and pBabe-Puro vector ^25^ plasmids were independently transfected into Phoenix amphotropic helper cells ^23^ using Lipfectamine LTX (Life Technologies) per the manufacturer’s instructions to generate defective retroviruses. The media was changed after 16 hours to DMEM/10% fetal bovine serum. After 24 hours the media containing the virus was removed and polybrene was added to 4 μg/mL. This viral media was filtered with 0.45 μm polyethersulfone filters (Thermo Scientific) and added to Scp-21 cells. This infection media was removed after 24 hours and selection in puromycin (10 μg/mL; Sigma Aldrich) was started 24 hours later. These Scp-21 cells expressing Fra-1 or control vector were maintained in DMEM supplemented with 10% fetal bovine serum and 5 μg/mL puromycin.

Scp-2 and Scp-21 cells used to measure protein degradation with cycloheximide were plated at 2×10 ^6^ cells in a 6 cm plate overnight. Plates were then treated with cycloheximide (10 μg/mL) for the indicated times.

### RNA purification and cDNA

RNA was purified from adherent cells with Trizol Reagent (Life Technologies) per the manufacturer’s instructions. RNA was reverse transcribed with the ImProm-II Reverse Transcriptase (Promega) according to the manufacturer’s instructions with random hexamer primers (Integrated DNA Technologies).

**Figure 1.**
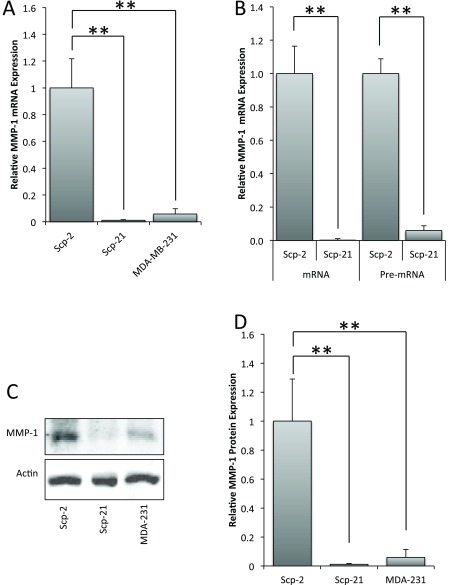
Differential expression of MMP-1 in MDA-MB-231 derivative cell lines. **A**) qPCR of MMP-1 mRNA expression in Scp-2 (high metastatic potential), Scp-21 (non-metastatic) and MDA-MB-231 (low metastatic) cells. **B**) qPCR of MMP-1 mRNA and pre-mRNA in Scp-2 and Scp-21 cells. Mean relative values +/- standard deviation from three independent experiments are shown. **C**) Immunoblot with anti-MMP-1 antibody of whole cell lysates from Scp-2, Scp-21, and MDA-MB-231 cells. Anti-actin antibody served as a loading control. **D**) Mean MMP-1 protein band intensity from immunoblots +/- standard deviation from three independent experiments. **, p < 0.005 for two-tailed t-tests.

In Figure 1B where pre-mRNA was measured with intronic primers and signal from contaminating genomic DNA can be problematic, samples measured by quantitative RT-PCR were treated with DNase I (Sigma) per the manufacturer’s instructions.

### Quantitative reverse transcription-polymerase chain reaction (qPCR)

qPCR was performed with standard protocols with the StepOne Plus System (Life Technologies) with Power SYBR master mix (Life Technologies) per the manufacturer’s instructions. Briefly, cDNA samples were combined with master mix and primers (final concentration 0.5 μM; shown in Table 1). Expression was normalized to 18S rRNA expression. Standard deviations were calculated from three independent experiments. p-values were determined by Student’s two-tailed t-tests with significance thresholds as labeled.

**Table 1.** Oligonucleotide primers used for quantitative RT-PCR.

RNA	Sequence (5′ – 3′)	
18S	F	TCGAGGCCCTGTAATTGGAAT
	R	CCCTCCAATGGATCCTCGTTA
MMP-1	F	CCTAGTCTATTCATAGCTAATCAAGAGGATGT
	R	AGTGGAGGAAAGCTGTGCATAC
MMP-1 Pre-mRNA	F	GCTGTGCTGTTACCCTAGTCCCT
	R	GGCAGCCAATCCCTTTGTT
c-Fos	F	CTGGCGTTGTGAAGACCATGA
	R	CCCTTCGGATTCTCCTTTTCTC
FosB	F	AGCAGCAGCTAAATGCAGGA
	R	TTTTGGAGCTCGGCGATCT
Fra-1	F	CCGGGCATGTTCCGAGACTT
	R	ACTCATGGTGTTGATGCTTGGCAC
Fra-2	F	AACTTTGACACCTCGTCCCG
	R	CCAGGCATATCTACCCGGAAT
c-Jun	F	AGATGAACTCTTTCTGGCCTGCCT
	R	ACACTGGGCAGGATACCCAAACAA
JunB	F	AGTCCTTCCACCTCGACGTTTA
	R	TGAATCGAGTCTGTTTCCAGCA
JunD	F	GACAAGCTTATGGAAACACCCTTCTACGG
	R	CCGGGATCCTCAGTACGCGGGCACCTGG

Primers (Integrated DNA Technologies) were designed using Primer Express (Life Technologies) with standard parameters. Primer sequences for the human genes are shown in Table 1.

### Conservation mapping

The matrix metallopeptidase-1 (MMP-1) promoter was analyzed with the UCSC Genome Browser ^26^. Analysis was performed on the following tracks: 1) Base Position, 2) Human mRNAs, 3) Placental Mammal Conservation by PhastCon with all 23 species, and 4) Vertebrate Conservation by PhastCon with all 46 species.

### Luciferase assays

All constructs were made using the pGL3-Basic promoter backbone with inserts at the BglII and HindIII sites of the multiple cloning sequence. The MMP-1 promoter regions were amplified from human genomic DNA (Bioline). Amplified inserts spanned -819/+71, -514/+71, -174/+71, -810/-174, -172/-27, -115/-27, and -94/-27 bases from the transcription start site. Shorter promoter inserts were annealed from oligonucleotide sequences as shown in Table 2.

**Table 2.** Oligonucleotide sequences used to construct luciferase reporter genes. The indicated forward and reverse oligonucleotides for MMP-1 promoter fragments were annealed and cloned upstream of the c-Fos minimal promoter in a pGL3 Basic backbone. The AP1, PEA3 and HoxA5 point mutants were made in the -107/-57 fragment. The 3xAP1 and 3xPEA3 sites were cloned upstream of the c-Fos minimal promoter.

**-107/-57 MMP-1 promoter fragment**	
F	AGCTGTCTATTCATAGCTAATCAAGAGGATGTTATAAAGCATGAGTCAGACAGCCT
R	GATCAGGCTGTCTGACTCATGCTTTATAACATCCTCTTGATTAGCTATGAATAGAC
**-74/-27MMP-1 promoter fragment**	
F	AGCTAGCATGAGTCAGACAGCCTCTGGCTTTCTGGAAGGGCAAGGACTCTCGTAC TCAGTCTGTCGGAGACCGAAAGACCT TCCCGTTCCTGAG
R	GATCCTCAGGAACGGGAAGGTCTTTCGGTCTCCGACAGACTGAGTACGAGAGTC CTTGCCCTTCCAGAAAGCCAGAGGCTGTCTGACTCATGCT
**-59/-27MMP-1 promoter fragment**	
F	AGCTCCTCTGGCTTTCTGGAAGGGCAAGGACTCTCGTACTCAGTCTGTCGGAGAC CGAAAGACCT TCCCGTTCCTGAG
R	GATCCTCAGGAACGGGAAGGTCTTTCGGTCTCCGACAGACTGAGTACGAGAGTC CTTGCCCTTCCAGAAAGCCAGAGG
**AP-1 point mutant**	
F	AGCTGTCTATTCATAGCTAATCAAGAGGATGTTATAAAGCATGCCACAGACAGCCT
R	GATCAGGCTGTCTGTGGCATGCTTTATAACATCCTCTTGATTAGCTATGAATAGAC
**PEA3 point mutant**	
F	AGCTGTCTATTCATAGCTAATCAAGATCTTGTTATAAAGCATGAGTCAGACAGCCT
R	GATCAGGCTGTCTGACTCATGCTTTATAACAAGATCTTGATTAGCTATGAATAGAC
**HoxA5 point mutant**	
F	AGCTGTCTATTCATAGCATGCCAAGAGGATGTTATAAAGCATGAGTCAGACAGCCT
R	GATCAGGCTGTCTGACTCATGCTTTATAACATCCTCTTGGCATGCTATGAATAGAC
**3xAP-1**	
F	AGCTCATGAGTCAGACATGAGTCAGACATGAGTCAGA
R	GATCTCTGACTCATGTCTGACTCATGTCTGACTCATG
**3xPEA3**	
F	AGCTAATCAAGAGGATGTTAAGCTAATCAAGAGGATGTTAAGCTAATCAAGAGG ATGTTA
R	GATCTAACATCCTCTTGATTAGCTTAACATCCTCTTGATTAGCTTAACATCCTCTT GATT

The -819/-174, -172/-27, -115/-27, -94/-27, -74/-27, and -59/-27 inserts and synthetic promoters were added upstream of a c-Fos minimal promoter insert ^27^ in the pGL3-Basic backbone. -819/+71 AP-1 point mutations were made by PCR driven overlap extension ^28^. pRL-SV40P with the SV40 promoter driving Renilla luciferase ^29^ served as an internal control. pCMV-Luciferase ^30^ served as a control.

The luciferase plasmids were transfected into cells using Lipofectamine 2000 (Life Technologies) per the manufacturer’s instructions. Cells were lysed in passive lysis buffer (Promega) 16 hours post transfection and analyzed using the Dual-Luciferase Reporter Assay System (Promega) per the manufacturer’s instructions with a 20/20 luminometer (Turner Biosystems) for a 10-second interval measurement. Mean and standard deviation for the ratio of firefly-luciferase to renilla-luciferase signals were calculated from three independent experiments. p-values were determine by Student’s two-tailed t-tests, with significance thresholds as indicated.

### Immunoblot analysis

Whole cell lysates were prepared with RIPA buffer (50 mM Tris, 150 mM NaCl, 0.1% SDS, 0.5% sodium deoxycholate, 1% Triton-100, 1 mM DTT, 1 mM PMSF, Protease Inhibitor Cocktail III [1:200; Calbiochem], pH 7.6). After 10 minutes at 4°C, the lysates were centrifuged at 20,000 g for 15 minutes at 4°C, and lysate supernatants were normalized for protein levels with BCA Assays (Pierce) per the manufacturer’s instructions. Normalized lysates were separated by SDS-polyacrylamide gel electrophoresis (PAGE), transferred onto Trans-Blot transfer medium (Bio-Rad), and immunoblotted with primary antibody at 4°C for 16 hours. Antibodies used were against Fra-1 (rabbit polyclonal; sc-605X), MMP-1 (goat polyclonal; sc-12348), JunD (rabbit polyclonal; sc-74X), c-Jun (rabbit polyclonal; sc-1694X), HSP-90 (mouse monoclonal; sc-101494) and Actin (goat polyclonal; sc-1616) from Santa Cruz Biotechnology. Dilutions of 1:1000 of these antibodies were used in immunoblots. Membranes were then washed three times with Tris-buffered saline (TBS) and incubated with secondary antibody at a 1:10,000 dilution for one hour. Secondary antibodies used were: Goat anti-Rabbit IRDye 800CW, Goat anti-Rabbit IRDye 680LT, and Donkey anti-Goat IRDye 800CW from LiCor. Membranes were then washed three times with TBS. Membranes were measured for fluorescence with an Odyssey infrared imager (LiCor). Means and standard deviations were calculated from Odyssey quantitation of specific band intensities in three independent experiments. p-values were determined by Student’s two-tailed t-tests with significance thresholds as indicated.

### siRNA treatment

Double stranded siRNA duplexes (Integrated DNA Technologies, Sigma-Aldrich), as indicated in Table 3, were transfected with RNAiMax Lipofectamine transfection reagent (Life Technologies) per the manufacturers instructions. Duplexes were designed as shown in Table 3.

**Table 3.** siRNA oligonucleotide sequences. The two oligonucleotides shown for each gene were annealed for use as siRNA. The Manufacture and catalog number for each are indicated.

Name	Sequences	Catalog number	Manufacturer
Control	CGUUAAUCGCGUAUAAUACGCGU AUACGCGUAUUAUACGCGAUUAACGAC	DS NC1	Integrated DNA technologies
dsiRNA-Fra-1 A	GGCGGAGACUGACAAACUGGAAGAT GUCCGCCUCUGACUGUUUGACCUUCUA	HSC.RNAI.N005438.12.1	Integrated DNA technologies
dsiRNA-Fra-1 B	CCACUUUACCCACCUAGAACACUAA ACGGUGAAAUGGGUGGAUCUUGUGAUU	HSC.RNAI.N005438.12.2	Integrated DNA technologies
dsiRNA-JunD A	CGAGUCCACAUUCCUGUUUGUAATC AUGCUCAGGUGUAAGGACAAACAUUAG	HSC.RNAI.N005354.12.1	Integrated DNA technologies
dsiRNA-JunD B	GCCGACGAGGCUCACAGUUCCUCUAC UGCGGCUGCUCGAGUGUCAAGGAGAUG	HSC.RNAI.N005354.12.3	Integrated DNA technologies
dsiRNA-c-Jun A		SAS_Hs02_00333461	Sigma-Aldrich
dsiRNA-c-Jun B		SAS_Hs01_00150279	Sigma-Aldrich

### Electrophoretic mobility shift assays (EMSA)

Nuclear extracts were made from 4×10 ^7^ cells grown on four 15 cm plates. Cells were washed with PBS at 4°C, and scraped into 3 mL of PBS. Samples were centrifuged at 400 g for 1 minute at 4°C in a J6B centrifuge (Beckman). The cell pellets were resuspended in 4 mL of Buffer A (10 mM Tris, 1.5 mM MgCl _2_, 10 mM KCl, 0.4 mM DTT, 0.04 mM PMSF, pH 7.9) and incubated for 10 minutes at 4°C. Samples were dounced 50 times with a type B 15 mL glass douncer (Kontes Glassware Co.). Dounced samples were centrifuged at 400 g for 10 minutes at 4°C in the J6B centrifuge. The nuclear pellets were resuspended in 300 μl Buffer C (20 mM Tris, 0.3 M KCl, 1.5 mM MgCl _2_, 25% Glycerol, 0.2 mM EDTA, 0.5 mM DTT, 0.5 mM PMSF, pH 7.9) and rotated at 4°C for 30 minutes. Samples were centrifuged at 20,000 g for 15 minutes at 4°C. Nuclear extract supernatants were then removed, normalized for total protein levels by BCA Assays (Pierce) and used for DNA binding reactions.

Probes and competitors for DNA binding assays were made with annealed complementary oligonucleotides (Integrated DNA Technologies), as shown in Table 4.

**Table 4.** Double stranded oligonucleotide probes used for Electrophoretic Mobility Shift Assays. The Forward and Reverse oligonucleotides were annealed for each probe.

**MMP-1 Probe (with AP-1 consensus site)**	
F	AGCTGTCTATTCATAGCTAATCAAGAGGATGTTATAAAGCATGAGTCAGACAGCCT
R	GATCAGGCTGTCTGACTCATGCTTTATAACATCCTCTTGATTAGCTATGAATAGAC
**Non-specific competitor:**	
F	TGTCGAATGCAAGCCACTAGAA
R	TTCTAGTGGCTTGCATTCGACA
**Probe with mutant AP-1 site:**	
F	AGCTGTCTATTCATAGCTAATCAAGAGGATGTTATAAAGCATGCCACAGACAGCCT
R	GATCAGGCTGTCTGTGGCATGCTTTATAACATCCTCTTGATTAGCTATGA ATAGAC

The annealed probes were end-labeled with γ- ^32^P-ATP (Perkin Elmer) and poly nucleotide kinase (New England Biolabs), per the manufacturer’s instructions, to a final concentration of 1 ng/µl. DNA binding reactions contained 5 μl nuclear extract (approximately 10 μg), 1 ng of ^32^P-labeled probe, 50 ng poly dI-dC, 250 ng of competitor (as indicated), and 2 μg antibody (in supershift experiments) for 30 minutes at room temperature with binding buffer (final concentration: 10 mM Tris HCl (pH 8.0), 50 mM KCl, 0.5 mM EDTA, 0.1% Triton-X 100, 12.5% Glycerol, 0.2 mM DTT). Samples were then loaded on a 5% polyacrylamide gel in 1/4× TBE, and run for 2.5 hours at 100 V with 1× TBE running buffer. The gel was then dried and exposed to x-ray film (Kodak) for 16 hours.

EMSAs for SRF were performed as above except with a high affinity SRF binding site, XGL, derived from the c-fos Serum Response Element ^31^.

### Chromatin immunoprecipitation (ChIP)

ChIP was performed as described ^32^, with minor modifications. Briefly, 4×10 ^7^ cells were crosslinked with 1% formaldehyde for 15 minutes at 25°C and quenched with 125 mM glycine. Crosslinked plates were lysed in RIPA buffer (as described in immunoblot methods above), sonicated with a Sonicator 3000 (Misonix) for 1 minute total, in 5 seconds on – 15 seconds off intervals, and centrifuged at 20,000 g for 15 minutes at 4°C. Lysates were normalized by BCA Assay (Pierce), per the manufacturer’s instructions, and immunoprecipitated with 2 μg of anti-Fra-1 antibody (Santa Cruz Biotechnology; Catalog #: sc-605) overnight rotating at 4°C. Protein-A agarose beads (7.5 μL) (Santa Cruz Biotechnology) diluted with 22.5 μL RIPA were added to purify immunoprecipitated protein for 90 minutes rotating at 4°C. The beads were washed three times in RIPA buffer and reconstituted in 200 μL elution buffer (70 mM Tris HCl pH 8.0, 1 mM EDTA, 1.5% SDS) for 10 minutes at 65°C. Beads were centrifuged at 1700 g for 1 minute at room temperature. The salt of the transferred supernatant was adjusted to a concentration of 200 mM NaCl and incubated for 5 hours at 65°C to reverse the crosslinks. DNA from ChIP samples was then purified with Qiaquik PCR Purification Kits (Qiagen) per the manufacturer’s instructions.

Purified DNA was measured by qPCR (as previously described in the qRT-PCR method) with primers shown in Table 5.

**Table 5.** Primer sequences used for chromatin immunoprecipitation experiments to detect binding at AP-1 or control sites in the MMP-1 and IL-6 genes.

**MMP-1**		
AP-1	F	TCTGCTAGGAGTCACCATTTCT
	R	ATAGAGTCCTTGCCCTTCCAG
Control	F	AGTGACTACCGCTCTGCTGTGT
	R	GTTCCGTCAGTCCTCATGGTT
**IL-6**		
AP-1	F	CTTCGTGCATGACTTCAGCTTT
	R	AGCGCTAAGAAGCAGAACCACT
Control	F	ATAGACGGATCACAGTGCACG
	R	GCAACGTAGACACTCCTGAACC

Samples were normalized to input DNA purified from reversed cross-linked input samples and measured through qPCR. Mean and standard deviations were calculated from three independent experiments. p-values were determine by Student’s two-tailed t-tests, with significance thresholds as indicated.

### Metabolic labeling

Cells (1×10 ^5^) were plated in 6 cm plates for labeling. After 16 hours, cells were washed twice with warm PBS and starved for 30 minutes at 37°C with 4 mL methionine and cysteine free DMEM (Life Technologies). Media was changed to 2 mL ^35^S-Translabel metabolic labeling reagent (100 μCi/mL; MP Biomedicals) in methionine and cysteine free DMEM and incubated at 37°C for the indicated times. Plates were washed twice with cold PBS, lysed in ice cold RIPA buffer, centrifuged at 20,000 g for 15 minutes at 4°C, and immunoprecipitated with 2 μg of anti-Fra-1 antibody (Santa Cruz Biotechnology; #sc-605) overnight rotating at 4°C. Protein-A agarose beads (Santa Cruz Biotechnology) were used to purify immunoprecipitated protein by incubation for 90 minutes at 4°C. Washed beads were reconstituted in SDS-PAGE sample buffer (described above) and boiled for 5 minutes. Boiled samples were centrifuged at 1700 g for 1 minute at 4°C, and resolved on 12% SDS-PAGE for 2.5 hours at 150 V. The gel was placed in fixative (50% methanol/10% acetic acid) for 30 minutes rocking at 25°C. The gel was then enhanced with Amplify Fluorographic Reagent (GE) for 30 minutes rocking at 25°C. After enhancement, the gel was dried and exposed to film (Kodak) for 5 days. Autoradiographs were quantitated by ImageJ software analysis. Means and standard deviations were calculated from three independent experiments. p-values were determine by Student’s two-tailed t-tests with significance thresholds as indicated.

### Soft agar assay

Soft agar plating of the cell lines was performed as described ^33^, with minor modifications. Briefly, 35-mm plates were coated with 1.5 mL 0.6% agar in DMEM. Cells (5×10 ^3^) were reconstituted in 1.5 mL 0.3% agar in DMEM, and plated on top of the 0.6% agar layer. Agar layers were then covered with 1.5 mL DMEM/10% Fetal Bovine Serum. Cells were grown for 21 days with the media being changed every 5 days. Colonies were stained with 0.005% Crystal Violet in water for one hour and counted. Mean colony number and standard deviation were calculated from three independent experiments. p-values were determined by Student’s two tailed t-tests with significance thresholds as indicated.

### Scratch-wound motility assays

Cells were grown to confluency and the monolayer was scratched and monitored by phase contrast microscopy. Cells were allowed to grow to 95% confluency and scratched with a pipette tip. Pictures of the cells were taken at 0 and 18 hours after the scratch. Images were taken at 100X magnification on a Nikon Diaphot 300 microscope. Triplicate images at each time point were used to count the number of cells that passed the scratch threshold. Mean and standard deviation were calculated from three independent experiments. p-values were determined by Student’s two-tailed t-tests, with significance thresholds as indicated.

## Results

### Correlation of MMP-1 expression with cell line metastatic potential

We analyzed microarray gene expression data from a set of 16 breast carcinoma cell lines with well-characterized metastatic potential ^14^ for a correlation between gene expression and metastasis. To identify genes that were specifically upregulated in cells with high bone or lung metastatic potential, we grouped cell lines as either highly metastatic to the bone, to the lung, or neither (i.e. with low metastatic potential) ^14^. We determined the ratio of average expression in highly metastatic cell lines (bone or lung) to the low metastatic cells ( Table 6; Supplementary Table 1). Microarray data from four highly metastatic bone cell lines were used, eight lung metastatic lines and four low or non-metastatic lines (described in Materials and methods). The highest differential expression was found for the MMP-1 gene. MMP-1 was expressed nearly an average of 100 fold more in bone metastatic cells than non-metastatic cells. Expression was also strongly higher in lung metastatic cells (27 fold), albeit with a weaker p value (0.056).

**Table 6.** The top five genes with greatest expression differential in genes of high metastatic potential and low metastatic potential in bone and lung. High bone metastatic cell lines are: 1833, Scp-2, Scp-25 and Scp-46. High lung metastatic cell lines are: 1834, 3481, 4142, 4173, 4175, 4180, Scp-3 and Scp-28. Low metastatic cells lines are: MDA-MB-231, Scp-6, Scp-21 and Scp-26. The gene expression values for these cell lines were used in a two-tailed t-test to calculate relative p-values. Genes with p-values over 0.06 were not included.

Gene symbol	Bone (High/Low)	p-Value	Lung (High/Low)	p-Value
Top bone genes				
MMP1	98.28	0.000241	26.63	0.056189
SPANX(A1/A2/B1/B2/C)	18.23	0.006807	22.04	0.005703
SPANXC	14.59	0.003343	14.92	0.007564
SPANX(B1/B2)	12.82	0.017419	15.82	0.008754
CXCR4	9.33	0.002383	0.14	0.052374
IL11	8.44	0.001599	3.38	0.155692
SRGN	5.75	0.000088	0.99	0.970525
Top lung genes				
SPARC	2.01	0.222907	104.03	0.058864
MMP1	98.28	0.000241	26.63	0.056189
SPANX(A1/A2/B1/B2/C)	18.23	0.006807	22.04	0.005703
SPANXC	14.59	0.003343	14.92	0.007564
SPANX(B1/B2)	12.82	0.017419	15.82	0.008754
KRT81	3.32	0.101475	13.49	0.001711
SOX4	4.67	0.038471	11.39	0.003905

We confirmed the microarray data for MMP-1 by measuring expression by quantitative RT-PCR (qPCR) in three cell lines with varying metastatic potential: MDA-MB-231 (low metastatic), and two MDA-MB-231 derived sub-lines: Scp-2 (highly metastatic) and Scp-21 (non-metastatic). Similar to what was found by microarray data, MMP-1 mRNA expression was 90 fold higher in Scp-2 cells than in Scp-21 cells and 17 fold higher than in MDA-MB-231 cells ( Figure 1A, and Data File 1). Other MDA-MB-231 derived cell lines tested with high metastatic potential (Scp-28) and non-metastatic (Scp-3), similarly had high and low MMP1 expression, respectively, when measured by qPCR (data not shown and Data File 1). Immunoblot analysis confirmed that MMP-1 protein levels were commensurate with mRNA expression ( Figure 1C and D, and Data File 1). These data indicate that MMP-1 is differentially regulated in cells with different metastatic potentials.

In order to test whether differential mRNA expression of MMP-1 is transcriptionally regulated, we used qPCR to measure the relative amounts of MMP-1 pre-mRNA. Pre-mRNA levels preceding splicing is a more direct indicator of transcription. Pre-mRNA levels of MMP-1 were also greatly elevated in Scp-2 metastatic cells compared to the non-metastatic Scp-21 cells, suggesting that this difference is due to changes in transcription ( Figure 1B, and Data File 1).

### Mapping of gene regulatory elements

**Figure 2.**
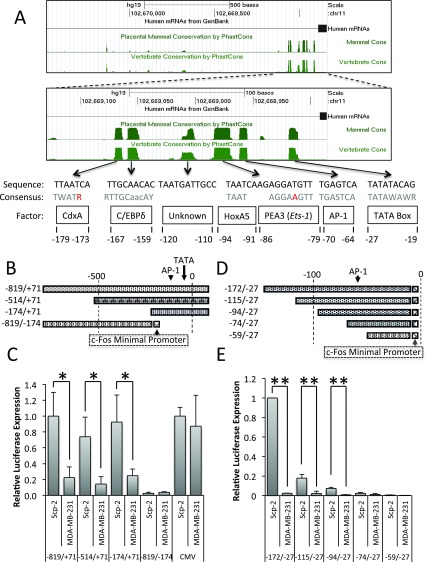
Regulatory regions and elements in the MMP1 promoter. **A**) Genome Browser analysis of placental mammalian and vertebrate conservation by PhastCon ^26^. Regions of conservation were compared to known transcription factor consensus sequences (shown in gray, with unmatched bases in red); numbers represent base position in reference to MMP-1’s transcription start site. **B**, **D**) Schematic of MMP-1 reporter constructs. **C**, **E**) Luciferase signal from Scp-2 and MDA-MB-231 cells transfected with the indicated reporter constructs. The signal was normalized to the Renilla luciferase levels from the co-transfected pRL-SV40P plasmid. The pCMV-luciferase construct was included as a control and its values were normalized to 1.0 for the Scp-2 cells. Its expression was approximately 10 times stronger than the MMP-1 -819/+71 luciferase reporter. The data shown represent the mean +/- standard deviation from three independent experiments. *, p < 0.05 for two-tailed t-tests.

We examined the human MMP-1 promoter for sequence conservation, and found blocks of conserved elements in the proximal promoter region ( Figure 2A). These conserved regions overlap consensus transcription factor binding sites that have previously been identified for the MMP-1 promoter ^34– 36^.

To determine if the MMP-1 promoter is sufficient to reproduce differential transcription in reporter assays, we inserted sections of the MMP-1 promoter in luciferase reporter constructs ( Figure 2B) and measured luciferase expression in Scp-2 and MDA-MB-231 cells (high and low metastatic cells, respectively). The MMP-1 promoter region from -819 to +71 was sufficient for five fold greater expression in the highly metastatic Scp-2 cells ( Figure 2C, and Data File 2).

In order to determine which region of the MMP-1 promoter was required for differential transcription of MMP-1 in Scp-2, Scp-21 and MDA-MB-231 cells, 5′ and 3′ deletions were made ( Figure 2B). Both -514/+71 and -174/+71 constructs were sufficient to drive significant differential expression, similar to -819/+71 ( Figure 2C). As a control, we used a CMV promoter-luciferase construct that gave similar expression in the two cell lines. These results suggest that key regulatory elements for differential expression are in the -174/+71 promoter region.

To further delineate the region required for differential expression, we designed a 3′ deletion -819/-174 construct. The -819/-174 MMP-1 region was inserted into a luciferase plasmid upstream of the c-Fos minimal promoter ( Figure 2B). The c-Fos minimal promoter includes the TATA box and transcription start site to give baseline expression. The -819/-174 construct was not able to drive significant expression ( Figure 2C). Together, the 5′ and 3′ deletion constructs identified the -174/+70 MMP-1 promoter region as necessary and sufficient for MMP-1 transcriptional regulation.

We also used the c-Fos minimal promoter with a series of 5′ MMP-1 promoter deletions to -27, to further isolate the region required for expression in -174/+70 ( Figure 2D). We found that the -94/-27 region was the minimal region required for differential expression between Scp-2 and MDA-MB-231 cells, with little differential expression seen with the -74 construct ( Figure 2E, and Data File 2). However, while the ratio of expression between Scp-2 and MDA-MB-231 was consistent among -172/-27, -115/-27 and -94/-27, overall expression was significantly lower in -94/-27 and -115/-27 compared to -172/-27, suggesting that there are positively acting regulatory elements between -74 and -172. These constructs showed that the -94 to -27 region was sufficient for differential expression by the MMP1 promoter.

**Figure 3.**
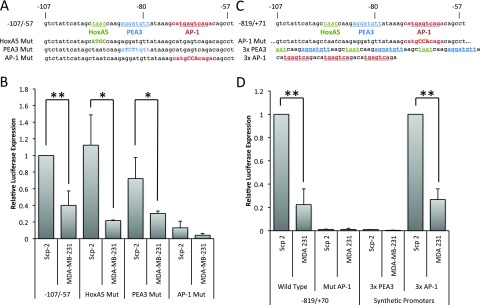
The AP-1 site of the MMP-1 promoter is necessary and sufficient for differential expression in Scp-2 and Scp-21 cells. **A**) Sequence of MMP-1 promoter regions inserted in reporter constructs. **B**) Luciferase signal from Scp-2 and MDA-MB-231 cells transfected with the indicated reporter constructs. The signal was normalized to the Renilla luciferase levels from the co-transfected pRL-SV40P plasmid. **C**) Sequences of the AP1 mutation in the -819/+71 reporter and of the 3× PEA3 and AP1 inserts. **D**) Luciferase signal from Scp-2 and MDA-MB-231 cells transfected with the indicated reporter constructs. The signal was normalized to the Renilla luciferase levels from the co-transfected pRL-SV40P plasmid. The 3x AP-1 construct values were normalized to 1.0 for the Scp-2 cells. Its expression was approximately three times stronger than the -819/+71 MMP-1 luciferase reporter. The data shown represent mean +/- standard deviation from three independent experiments. *, p < 0.05 ; **, p < 0.005 for two-tailed t-tests.

Having isolated a small regulatory region of the MMP-1 promoter, we sought to determine the specific transcription factor binding sites involved. Previous findings and conservation mapping pointed to several potential regulators in the -94/-27 region of the MMP-1 promoter: HoxA5, PEA3, and AP-1 ( Figure 2A) ^37, 38^. To determine which, if any, of these sites are required for regulation, synthetic promoters were made with the region that contains the three consensus sites, -107 to -57, upstream of the c-Fos minimal promoter ( Figure 3A). The -107/-57 region drove significantly higher expression in Scp-2 than in MDA-MB-231 cells ( Figure 3B, and Data File 3). This differential expression was lower than with the constructs used in Figure 2E suggesting that sequences flanking the -107 to -57 region can modulate the induction. Nevertheless, these reporters allowed us to check this minimal region for the role of regulatory elements. Point mutations were made to each of the three conserved consensus regions ( Figure 3A). Among them, only the AP-1 site mutation significantly decreased expression and decreased differential expression. While Scp-2 cells did have greater luciferase expression than MDA-MB-231 cells for the AP1 mutant, this low level was variable and the difference was not statistically significant ( Figure 3B).

To confirm that the AP-1 site is required for expression in the context of the fuller promoter, we created a -819/+71 MMP-1 promoter construct with point mutations in the AP-1 consensus site ( Figure 3C). These point mutations were sufficient to completely abrogate luciferase expression in both Scp-2 and MDA-MB-231 cells ( Figure 3D, and Data File 3). The AP1 site alone was not sufficient to drive expression in Scp-2 cells as the site is present in the -74/-27 construct that was not expressed ( Figure 2E). To determine whether multiple copies of the AP-1 site were sufficient, we made a synthetic promoter construct with a triple MMP-1 AP-1 consensus site and found that it gave a robust signal with significant differences between Scp-2 and MDA-MB-231 ( Figure 3C and D). The ratios of luciferase expression in Scp-2 versus MDA-MB-231 cells were similar with the triple AP-1 synthetic promoter and the -819/+71 region of MMP-1 ( Figure 3D). In contrast, a triple PEA3 site did not drive luciferase expression, suggesting that it is not sufficient for differential expression ( Figure 3D). Together these experiments demonstrated that the AP-1 region of the promoter is both necessary and sufficient for differential transcriptional regulation of MMP-1 in Scp-2 and MDA-231 cell lines.

### Characterization of AP-1 family members in MDA-MB-231 derived cell lines

**Figure 4.**
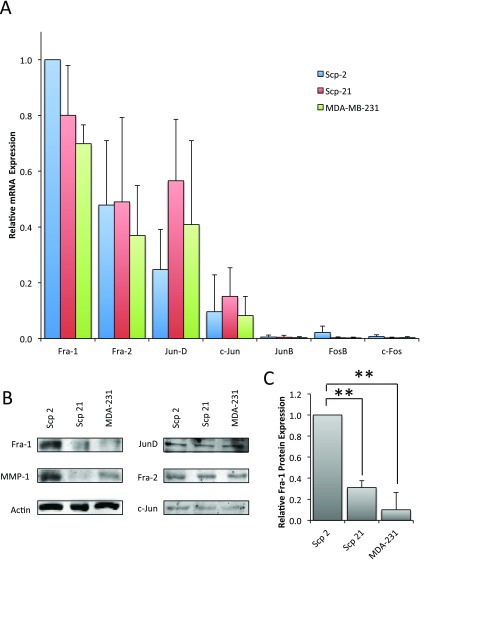
AP-1 family mRNA and protein expression. **A**) qPCR of AP-1 family member mRNA expression in Scp-2, Scp-21 and MDA-MB-231 cells. Mean relative values are shown +/- standard deviation from three independent experiments. **B**) Immunoblots with anti-AP-1 family antibodies of whole cell lysates from Scp-2, Scp-21, and MDA-MB-231 cells. Anti-MMP-1 is included for comparison and anti-actin antibody served as a loading control. **C**) Mean Fra-1 protein band intensity from immunoblots as in ( **B**) +/- standard deviation from three independent experiments. **, p < 0.005 for two-tailed t-tests.

The AP-1 consensus site is bound by a dimer of AP-1 family members reviewed in ^39^. There are seven AP-1 family genes: three Jun genes (c-Jun, JunB and JunD) and four Fos related genes (c-Fos, Fra-1, Fra-2, and FosB). Dimers are comprised of at least one Jun family member, but can be homo- or hetero-dimers ^40, 41^. To determine which AP-1 family members were expressed in Scp-2, Scp-21 and MDA-MB-231 cells, and would therefore be candidates for MMP-1 regulation, we performed qPCR in each of the cell lines. Fra-1, Fra-2 and JunD had the highest expression levels, with lower levels of c-Jun and nearly undetectable JunB, FosB and c-Fos ( Figure 4A, and Data File 4). However, unlike the differential mRNA expression seen for MMP-1 ( Figure 1A), all the detectable AP-1 family members had comparable mRNA expression among the different cell lines ( Figure 4A). While there was significant variability in expression levels in experimental repeats, and hence the relatively large error bars, there was no consistent difference in expression among the cell lines.

To explore whether AP-1 family member protein expression is consistent with their mRNA expression, we performed immunoblots. Specifically, we looked at Fra-1, Fra-2, c-Jun and JunD in Scp-2, Scp-21 and MDA-MB-231 cell lines. The remaining AP-1 family members, c-Fos, FosB and JunB, that were not expressed at the mRNA level were not considered further. Interestingly, contrary to Fra-1 mRNA expression levels, Fra-1 protein levels were significantly higher in Scp-2 cells than Scp-21 and MDA-MB-231 cells ( Figure 4B and C, and Data File 4). However, there was no significant difference in protein expression levels of Fra-2, JunD or c-Jun. These results suggest the possibility that differences in Fra-1 protein expression in Scp-2, Scp-21 and MDA-MB-231 cell lines are responsible for regulation of MMP-1 transcription.

**Figure 5.**
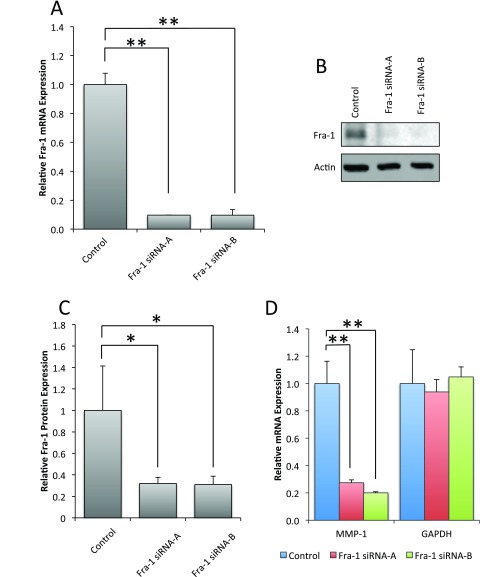
Fra-1 is required for MMP-1 mRNA expression in Scp-2 cells. **A**) Scp-2 cells were transfected with control or two independent siRNA duplexes for Fra-1. Fra-1 mRNA levels were measured by qPCR. **B**) Scp-2 cells transfected with control or Fra-1 siRNA duplexes were immunoblotted with anti-Fra-1 antibodies. Anti-actin antibody served as a loading control. **C**) Mean Fra-1 band intensity from immunoblots as in ( **B**) from three independent experiments. **D**) qPCR of MMP-1 and GAPDH control mRNA expression of Scp-2 cells transfected with control or Fra-1 siRNA duplexes. In **A**, **C** and **D**, mean relative values are shown +/- standard deviation from three independent experiments. *, p < 0.05; **, p < 0.005 for two-tailed t-tests.

To test the hypothesis that Fra-1 regulates MMP-1, we inhibited expression of Fra-1 in Scp-2 cells with short interfering RNAs (siRNA). Two siRNA duplexes decreased Fra-1 mRNA expression by over 80% ( Figure 5A) and Fra-1 protein levels by about 70% ( Figure 5B and C, and Data File 5). This inhibition greatly reduced MMP-1 mRNA expression ( Figure 5D, and Data File 5), supporting Fra-1’s role in MMP-1 regulation.

**Supplemental Figure S1.**
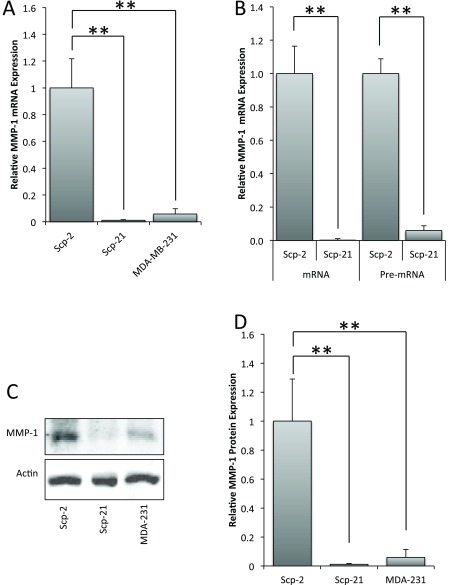
JunD depletion with siRNAs has no effect on MMP-1 mRNA expression. **A**) Immunoblot with anti-JunD antibodies of Scp-2 cells transfected with control or siRNA duplexes for JunD. **B**) Mean JunD band intensity +/- standard deviation for immunoblots from three independent experiments as in ( **A**). **C**) qPCR of JunD, MMP-1 and Fra-1 in Scp-2 cells transfected with control or siRNA duplexes for JunD. Mean relative values are +/- standard deviation from three independent experiments are shown. **, p < 0.005 for two-tailed t-tests.

Though other AP-1 family members were not differentially expressed in the Scp-2, Scp-21 and MDA-MB-231 cell lines, we sought to determine which other AP-1 family members were required for MMP-1 expression. As JunD is the most strongly expressed Jun family member in these cells, we first inhibited its mRNA expression with siRNA duplexes ( Supplementary Figure 1). However, despite efficient reduction in JunD levels, this inhibition did not have an effect on MMP-1 mRNA expression ( Supplementary Figure 1).

We had difficulty efficiently depleting Fra-2 and c-Jun with siRNAs, perhaps because mRNA expression of these genes was relatively low. As such, partial inhibition of c-Jun and Fra-2 had no statistically significant impact on MMP-1 (data not shown). Therefore, it was not possible for us to assess whether c-Jun, or c-Jun acting redundantly with JunD, were required for MMP-1 expression. Nevertheless, the requirement of the AP-1 site in the MMP1 promoter and depletion of Fra-1 clearly show that this factor is required for expression of MMP1 in the metastatic MDA-MB-231 derived cells. The differential expression of Fra-1 protein levels suggests that this mechanism may at least partially account for differences in MMP1 expression.

### Binding of Fra-1 to the MMP-1 AP-1 site

**Figure 6.**
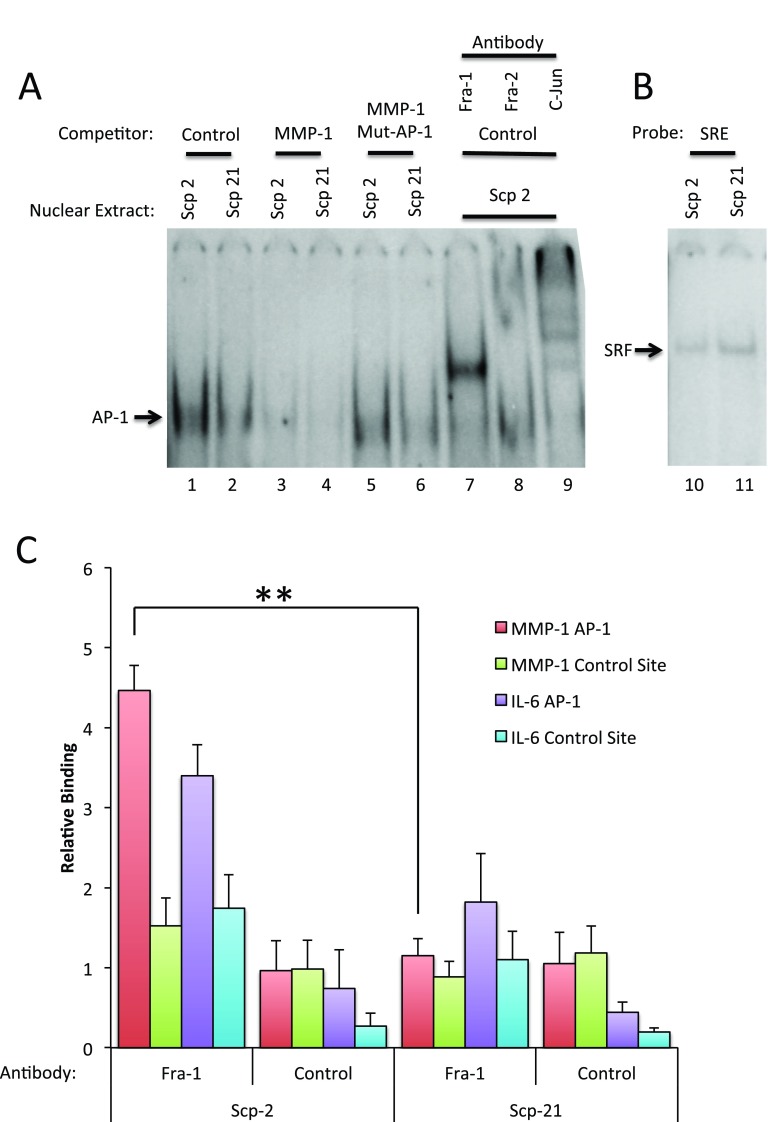
Fra-1 binds site of the MMP-1 promoter. **A**) *In vitro* binding. Electrophoretic mobility shift assay (EMSA) with the MMP-1 AP-1 site. Scp-2 and Scp-21 nuclear extracts were incubated with a ^32^P-end labeled MMP-1 promoter double-stranded oligonucleotide probe spanning the AP-1 consensus site (-107 to -57 bases relative to the transcription start site). Nonspecific competitor, unlabeled, and point mutant AP-1 site oligonucleotides were added in 250 fold excess of the probe. The final three lanes included anti-AP-1 family member antibodies. **B**) Control EMSAs were preformed as in A except with a Serum Response Element probe to detect SRF binding. **C**) *In vivo* binding. Chromatin immunoprecipitation with Scp-2 and Scp-21 cells immunoprecipated with anti-Fra-1 antibody or mock antibody control. The immunoprecipitated DNA from the samples was measured by qPCR for binding of Fra-1 to the MMP-1 AP-1 promoter site, an upstream non-AP-1 control MMP-1 site, the IL-6 gene AP-1 site, or an upstream non-AP-1 control IL-6 site. The data shown represent the mean fold over control DNA values +/- standard deviation for three indendent experiments. **, p < 0.005 for two-tailed t-tests.

As previous experiments showed that Fra-1 was required for MMP-1 expression, we confirmed protein binding *in vitro* to the MMP-1 AP-1 site in the highly metastatic Scp-2 and non-metastatic Scp-21 cells using electrophoretic mobility shift assays (EMSA). The -107 to -57 region of the MMP-1 promoter, containing the AP-1 consensus sequence, was used as a probe for binding with nuclear extracts from Scp-2 and Scp-21 cells. Specific binding was observed ( Figure 6A lanes 1 and 2) which was competed by excess non-labeled competitor (lanes 3 and 4). Mutations in the AP-1 binding site abolished this competition, suggesting that the band is indeed AP-1 (lanes 5 and 6).

Interestingly, a stronger AP-1 complex was detected in the highly metastatic Scp-2 cells compared with the low metastatic Scp-21 cells ( Figure 6A, compare lanes 1 and 2). This is consistent with higher Fra-1 protein expression in Scp-2 cells and higher expression of MMP1 ( Figure 4B and C). As a control for the similarity of the nuclear extracts of the two cell lines, we examined binding of the transcription factor SRF to a Serum Response Element (SRE) probe and found no significant difference ( Figure 6B).

To determine which proteins in the nuclear extracts were present in the bound band, we used antibodies specific for AP-1 family members. Anti-Fra-1 antibody supershifted the band ( Figure 6A, lane 7), indicating that Fra-1 is a major component of the bound complex. In contrast, Fra-2 antibodies had little effect (lane 8). Antibodies to c-Jun strongly shifted the complex into multiple bands, suggesting that it too is in the complex (lane 9). We did not observe a shift with antibodies to JunD, however the antibodies may be ineffective for supershifts (data not shown). These EMSA experiments support the conclusion that Fra-1 and c-Jun are the predominant members of the AP-1 complex bound to the MMP-1 site.

To show Fra-1 binding and regulation of the MMP-1 promoter *in vivo*, we performed chromatin immunoprecipitation (ChIP) experiments in Scp-2 and Scp-21 cells. Antibodies to Fra-1 demonstrated higher binding to the MMP-1 promoter in Scp-2 cells than Scp-21, consistent with the relative MMP-1 expression in these cells ( Figure 6C, and Data File 6). A similar, though slightly weaker, difference was seen at a known AP-1 binding site in the IL-6 gene ( Figure 6C) ^42, 43^. Background signal was seen at distal control sites in the MMP-1 and IL-6 genes or with a non-specific control antibody.

### Fra-1 regulation is translational

Since Fra-1 is required for MMP-1 expression and binds preferentially to the MMP-1 promoter in Scp-2 cells, we analyzed Fra-1 regulation. As shown Figure 4, Fra-1 mRNA levels did not vary significantly among the metastatic variant cell lines, while Fra-1 protein levels were higher in Scp-2 cells. To better understand the post-transcriptional regulation of Fra-1, we analyzed Fra-1 protein degradation and translation.

**Figure 7.**
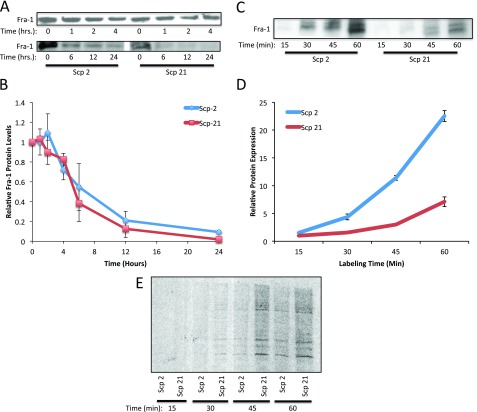
Regulation of Fra-1 protein translation. **A**) Fra-1 protein stability. Scp-2 and Scp-21 cells were treated with cycloheximide and whole cell lysates collected at the indicated times post-treatment. Immunoblots are shown with anti-Fra-1 antibody of representative experiments. **B**) The mean relative Fra-1 band intensities from immunoblots as in ( **A**) from three independent experiments +/- the standard deviation are shown. **C**) ^35^S-metabolic labeling of Fra-1. Cells were depleted of cysteine and methionine for 30 minutes and labeled with ^35^S-cysteine and -methionine for the indicated times and immunoprecipitated. **D**) Fra-1 protein levels as in ( **C**) were quantified and normalized to total protein labeling. The mean band intensity +/- standard deviation from three independent experiments. **E**) Control for general protein synthesis. Autoradiograph of total protein on an SDS-polyacrylamide gel from cells depleted of cysteine and methionine for 30 minutes and labeled with ^35^S-cysteine and -methionine for the indicated times.

We first measured the degradation rate by blocking new protein translation using the protein synthesis inhibitor cycloheximide. By measuring protein levels over time, without de-novo translation, we could compare degradation rates of Fra-1 in Scp-2 and Scp-21 cells. We compared Fra-1 levels at either 0 to 4 or 0 to 24 hour intervals ( Figure 7A). While levels were somewhat variable in specific experiments (as seen in Figure 7A) we quantified the results from three repetitions. We found that Fra-1 protein was more abundant in Scp-2 than Scp-21 cells, as previously seen, and when we normalized to the starting relative levels in each cell line, we found that there was no significant difference in the stability of Fra-1 in these two cell lines ( Figure 7B, and Data File 7).

With no difference in Fra-1 protein degradation or mRNA levels, we measured Fra-1 translation rates. Scp-2 and Scp-21 cells were labeled with ^35^S-labeled amino acids to measure amino acid incorporation into proteins over a one-hour interval. Immunoprecipitation of Fra-1 showed that its translation was significantly higher in Scp-2 cells than Scp-21 cells ( Figure 7C and D, and Data File 7). There was a slightly higher general protein synthesis in the Scp-21 cells as seen by running the total ^35^S-labeled cell lysates on an SDS-polyacrylamide gel ( Figure 7E). This is the opposite direction as seen for Fra-1 protein synthesis. Together with the lack of change in Fra-1 protein stability and the approximate half-life of 5 hours ( Figure 7B), these results suggest that Fra-1 is regulated at the level of protein translation.

### Stable expression of Fra-1 in Scp-21 cells increases MMP-1 expression, motility and anchorage independent growth

**Figure 8.**
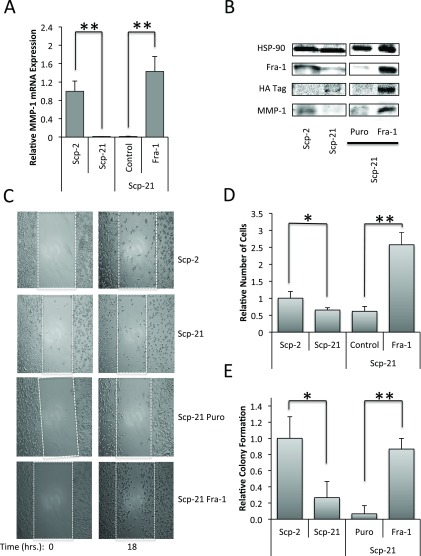
Effects of Fra-1 over expression in Scp-21 cells. **A**) qPCR of MMP-1 in Scp-2, Scp-21, Scp-21 control vector (Puro), and Scp-21 cells stably expressing HA-tagged Fra-1. Mean relative values are +/- standard deviation from three independent experiments. **B**) Immunoblots of the indicated cell lines with anti-MMP-1, anti-Fra-1 and anti-HA antibodies. Anti-HSP-90 served as a loading control. **C**) Effect on cell motility. Scratch-wound assays of the indicated cell lines were performed with cell motility measured 0 and 18 hours after the scratch. Cells were grown to 95% confluency and scratched with a pipette tip. Images were taken at 100X magnification. **D**) Quantitation of the number of cells crossing the initial scratch threshold at 18 hours. The means of three fields in three independent experiments +/- standard deviation are shown. **E**) Anchorage independent growth. The indicated cell lines were grown in soft agar for 21 days. The means +/- standard deviation of the relative number of colonies formed in three independent experiments are shown. *, p < 0.05. **, p < 0.005 for two-tailed t-tests.

To determine the effect of higher Fra-1 expression in non-metastatic cells, we created Scp-21 cells that stably express Fra-1. Control Scp-21 cells that stably express a control vector, have low Fra-1 protein expression, while the cells infected with a Fra-1 retrovirus expressed high levels, several fold higher than that in Scp-2 cells ( Figure 8B). We found that higher Fra-1 expression resulted in higher MMP1 mRNA and protein expression, suggesting that higher levels of Fra-1 are sufficient for increased MMP1 expression ( Figure 8A and B, and Data File 8).

Since increased Fra-1 protein expression correlated with increased MMP-1 expression and metastasis, we tested whether it is sufficient to drive properties of metastatic cells, in particular cell motility and anchorage independent growth. A scratch assay, or wound healing assay, was used to measure cell motility. We found that Scp-2 had greater motility than Scp-21 cells, and that Scp-21 cells expressing Fra-1 had significantly greater motility than vector control Scp-21 cells ( Figure 8C and D, and Data File 8). Surprisingly, Scp-21 cells expressing Fra-1 had even greater motility than highly metastatic Scp-2 cells. Therefore, Fra-1 expression increases motility.

Non-metastatic cells often grow poorly in soft agar, while metastatic cells can display increased anchorage independent growth ^44, 45^. Similar to the pattern seen in motility assays, Scp-2 showed significantly greater growth in soft agar than Scp-21 cells ( Figure 8E, and Data File 8). Interestingly, Scp-21 cells expressing Fra-1 greatly increased the growth of the cells in soft agar, similar to the Scp-2 levels. These results indicate that increased Fra-1 expression in Scp-21 cells was sufficient for increased anchorage independent growth.

Data files for figures 1-8 Data file 1. Data for figures 1A, B and D and data not shown. A, B) Relative mRNA expression from the indicated cell lines is shown for three replicates from independent mRNA isolations. The numbers reflect qPCR delta Ct numbers using 18S rRNA Ct values. The values were scaled to an average of 1.0 for Scp-2 cells. The primers used are shown in Materials and Methods. D) Relative MMP-1 protein expression. Three immunoblots were performed with anti-MMP1 sera and lysates of the indicated cell lines as in figure 1C. The MMP-1 band intensities were quantified using the Odyssey infrared imager (Li-Cor) and the software provided by the manufacturer. The intensities were scaled to the average intensities of MMP-1 in Scp-2 cells. Data not shown) Relative MMP-1 expression in other MDA-MB-231 single cell population cell lines as in figure 1A. Values are from two independent replicates.
Data file 2. Data for figures 2C and E. Relative luciferase activity is shown for three replicates. Each replicate is the average of duplicate determinations (i.e. six total points). The firefly luciferase values were divided by the Renilla luciferase values (from cotransfected plasmid pRLSV40P). In (C) the values were scaled to an average of 1.0 for the Scp-2 cells and the -819 to +71 MMP1 reporter, except for CMV which was scaled to its own values for Scp-2 cells. In (E) the values were scaled in each replicate to 1.0 for Scp-2 cells and the -172/-27 MMP1 reporter.
Data file 3. Data for figures 3B and D. Relative luciferase activity is shown for three replicates. Each replicate is the average of duplicate determinations (i.e. six total points). The firefly luciferase values were divided by the Renilla luciferase values (from cotransfected plasmid pRLSV40P). The values were scaled in each replicate to 1.0 for Scp-2 cells and the wild type reporter. For the synthetic reporter, the values were scaled to that of the 3X-AP1 site reporter.
Data file 4. Data for figures 4A and C. A) Relative mRNA expression from the indicated cell lines and genes is shown for three replicates from independent mRNA isolations. The numbers reflect qPCR Ct numbers using 18S rRNA Ct values. The values were scaled to a value of 1.0 for Scp-2 cells. The primers used are shown in Materials and Methods. C) Relative Fra-1 protein expression. Three immunoblots were performed with anti-Fra-1 sera and lysates of the indicated cell lines as in figure 4B. The Fra-1 band intensities were quantified using the Odyssey infrared imager (Li-Cor) and the software provided by the manufacturer. The intensities were scaled to the average values for Fra-1 protein in Scp-2 cells.
Data file 5. Data for figures 5A, C and D. A) Relative Fra-1 mRNA expression in Scp-2 cells treated with the indicated siRNAs. Expression determined by qPCR is shown for three replicates from independent mRNA isolations. The numbers reflect qPCR delta Ct numbers using 18S rRNA Ct values. The values were scaled to an average value of 1.0 for untreated Scp-2 cells. The primers used are shown in Materials and Methods. B) Relative Fra-1 protein expression. Three immunoblots were performed with anti-Fra-1 sera and lysates of Scp-2 cells treated with the indicated siRNAs as in figure 5B. The Fra-1 band intensities were quantified using the Odyssey infrared imager (Li-Cor) and the software provided by the manufacturer. The values were scaled to the average intensities of Fra-1 protein in Scp-2 cells. C) As in (A) exept that MMP-1 and GAPDH mRNA expression were measured by qPCR. The primers used are shown in materials and methods.
Data file 6. Data for figure 6C. Chromatin immunoprecipitation from the indicated cell lines and using anti-Fra1 or control (no antibody) was quantified by qPCR using the indicated primers. The binding values were divided by those of input DNA and are shown as percent of input. Values from three replicate chromatin immunoprecipitations are shown.
Data file 7. Data for figures 7B and D. B) Scp-2 or Scp-21 cells were treated with the protein synthesis inhibitor cycloheximide for the indictate times. The cell lysates were then immunoblotted with anti-Fra-1 antibodies as in figure 7A. The Fra-1 protein band intensities were quantified on the Odyssey infrared imager (Li-Cor). The values for three independent experiments are shown. The values were scaled to 1.0 for cells treated for 0 hours (i.e. untreated). D) Scp-2 and Scp-21 cells were metabolically labeled with 35S-methionine and –cysteine for the indicated times. Fra-1 protein was then immunoprecipitated with anti-Fra-1 antibodies, run on SDS-PAGE and exposed to film. The bands were quantified using ImageJ software. The values were scaled to the value of Fra-1 expression in Scp-21 cells at 15 minutes of labeling. Values for there independent replicates are shown.
Data file 8. Data for figures 8A, D and E. A) Relative MMP-1 mRNA expression in the indicated cell lines with no expression vector, control pBabepuro vector or Fra-1 expressing pBabFra-1 vector. Expression determined by qPCR is shown for three replicates from independent mRNA isolations. The numbers reflect qPCR delta Ct numbers using 18S rRNA Ct values. The values were scaled to an average value of 1.0 for untreated Scp-2 cells. The primers used are shown in Materials and Methods. D) Quantfication of cell motility as in Fig. 8C. The number of cells crossing the initial scratch after 18 hours were counted in three independent experiments. E) Quantification of anchorage independent growth in soft agar. The number of colonies growing after 21 days of plating in soft agar were counted in three independent experiments.

## Discussion

We have used a well defined system of metastatic cell variants to limit the heterogeneity of the samples and to provide a large number of closely related cell lines with variable metastatic potential ^14^. The cell lines are all derived from MDA-MB-231 breast carcinoma cells, either by selection of metastatic clones in mouse xenografts or by analysis of single cell clones (the Scp lines). The result that the Scp cell clones have vastly different, but reproducible, metastatic potentials suggests that the cells with these properties were pre-existing in the MDA-MB-231 cultures ^12, 14^. The analysis of gene expression in these cell lines yielded a list of genes correlated to metastatic potential. We have found that MMP-1 is among the most strongly elevated genes in cells with high metastatic potential and that this expression is transcriptionally regulated by Fra-1 interaction with the AP-1 site of the MMP-1 promoter. Fra-1 expression was also regulated, but at the level of protein translation.

### Minimal promoter region sufficient for differential expression

The mapping of sequence elements required for expression of MMP-1 in high vs. low metastatic cells did not reveal an element that was required in only one of the cell types. However, the AP-1 site was strongly required for expression in both cell lines. Furthermore, the synthetic triplicate AP-1 site promoter construct showed higher expression in the highly metastatic cells, demonstrating that it is sufficient to mediate higher expression. The single AP1 site in the MMP-1 promoter was not sufficient, suggesting that it normally requires the binding of other factors to function fully. Initial studies of phorbol ester induction of the MMP-1 promoter in fibroblasts had a similar result ^38^. Therefore, it is likely that additional factor binding to the -96/-74 region of the MMP-1 promoter is also required. However, transcription factors with known binding sites in that region, HoxA5 and PEA3, were not required. In addition, expression of the MMP-1 reporter gene was significantly greater with the -172/-27 region compared to the -115/-27 region. While this region was not required for differential expression, it is likely that additional factor binding in this region increases expression.

### Role of AP-1 in MMP-1 expression and metastasis

In line with our findings, AP-1 regulation of MMP-1 has been well studied in several systems ^18, 46, 47^ and AP-1 expression has been implicated in tumorigenesis ^22, 48, 49^. In particular, expression of Fra-1 has been shown to be correlated to plastic proliferative breast disorders ^21^ and aggressive breast cancer cells ^50^.

We have shown that Fra-1 is required for MMP-1 expression in the MDA-MB-231 derivatives. Fra-1 binds to the AP-1 consensus sequence as a heterodimer with a Jun protein ^51^. Therefore, a Jun protein should also be required for MMP-1 expression. However, depletion of the most highly expressed Jun protein, JunD, had no effect on MMP-1 expression. JunB could not be detected by immunoblotting and showed very low expression by qPCR. The final Jun protein, c-Jun, was detected by immunoblot and qPCR. However, five siRNA duplexes were unable to significantly reduce c-Jun expression (data not shown). Challenges inhibiting c-Jun expression may be due to its low levels or, alternatively, to a cell requirement for c-Jun expression—making c-Jun inhibition toxic to the cell. Due to the inability to strongly deplete c-Jun levels, we cannot determine whether it is required for MMP-1 expression or whether it fulfills a redundant requirement with JunD. It is also possible that there is a novel partner for Fra-1 in these cells.

We did not detect altered Fra-1 mRNA expression in the MDA-MB-231 cell variants, however higher mRNA expression has been observed in more metastatic ER negative cell lines when compared to less metastatic cells ^52, 53^. Differences in Fra-1 mRNA expression were also observed in breast cancer patients, where expression was higher in carcinomas compared with benign tumors ^21^. Thus, besides regulation of translation, as we have found here, alternative mechanisms to regulate Fra-1 mRNA expression may be important in some breast tumors.

### Translational regulation of Fra-1 regulates MMP-1

While Fra-1 mRNA levels were not significantly regulated in the MDA-MB-231 cell variants, immunoblots, EMSAs and chromatin immunoprecipitations showed that the metastatic variant Scp-2 cells have higher Fra-1 protein expression and higher DNA binding *in vitro* and *in vivo* to the MMP-1 AP-1 site. As Fra-1 was the only detectable AP-1 family factor that varied in the metastatic variants, this suggests that Fra-1 is responsible for the difference in MMP-1 expression. In addition, overexpression of Fra-1 in the low metastatic Scp21 cells increased MMP-1 expression, showing that higher Fra-1 expression is sufficient, as well as necessary, for elevated MMP-1 expression.

We found that Fra-1 protein levels were regulated by altered translation rates. There were little differences in the rates of protein degradation. However, short metabolic labeling showed increased synthesis of Fra-1 in the metastatic cell variant. Several studies have demonstrated that phosphorylation of Fra-1 by ERK1/2 increases its protein stability ^54– 57^. However, this mechanism does not appear to be functioning in the MDA-MB-231 cells, since we did not detect a change in degradation rates.

Recently, data from human cancer cell lines pointed to evidence of miRNA-34a regulation of Fra-1 and MMP-1 ^58, 59^. In breast cancers, miRNA-34a was inversely correlated to the metastatic potential of cell lines and tumor samples, but was not found to be different in paired tumor and normal breast tissue samples ^59^. Strikingly, expression of miRNA-34a in MDA-MB-231 cells reduced Fra-1 expression, matrigel invasion, and tumors in mouse xenografts. In addition, overexpression of Fra-1 rescued the suppressive effects of miRNA-34a on migration and invasion of MDA-MB-231 cells ^59^. While miRNA-34a regulation of Fra-1 is a strong hypothesis for MMP-1 regulation in MDA-MB-231 variants, a major difference is that we did not observe changes in Fra-1 mRNA expression as reported with miRNA-34a ^58, 59^. Separately, miRNA-143 was also found to target Fra-1 mRNA, suggesting that this and other miRNAs are also candidates for Fra-1 regulation ^60^. It will be interesting to determine which, if any, miRNA regulates Fra-1 translation in metastatic MDA-MB-231 variants.

Despite initial work supporting miRNA translational regulation without impact on mRNA levels ^61^, more recent evidence supports miRNA regulation of both protein and mRNA expression ^62, 63^. There are several examples of other genes being regulated by miRNA without discernable differences in mRNA levels ^64– 67^. As our experiments only show translational regulation, it is possible that Fra-1 is a case where miRNA regulation is entirely translational. Alternatively, it is possible that Fra-1 translation is regulated by a mechanism other than miRNA.

### Fra-1 induces metastatic properties

Stable expression of Fra-1 in non-metastatic MDA-MB-231 derivative cells led to greater MMP-1 expression, motility and anchorage-independent growth. This supports Fra-1 as an upstream regulator of MMP-1 and potentially of other genes required for increased metastatic properties. These results are in line with previous colon cancer data, correlating Fra-1 expression with escape from anoikis ^56^, and increased motility ^68^. In spontaneous murine mammary adenocarcinoma variants with different metastatic potential, Fra-1 expression also correlated to invasiveness ^69^. Transient transfections of Fra-1 in MDA-MB-231 and MCF-7 similarly increased matrigel cell invasion ^48^. Contrary to our findings, this overexpression had no impact on MMP-1 expression. In other experiments, however, overexpression of Fra-1 in MCF-7 cells increased cell invasion and MMP-1 expression ^52^. Recently, Fra-1 was also shown to be required for high metastasis in xenografts of a highly metastatic MDA-MB-231 derivative cell line ^70^.

### Potential functions of Fra-1 and MMP-1 in invasion and migration

Fra-1 has many direct and indirect targets ^71^. Fra-1 depletion in a highly metastatic MDA-MB-231 variant line altered the expression of 1,234 genes ^70^. Among these, E-cadherin has an inverse correlation with Fra-1, confirming previous results ^50^. Fra-1 expression has also previously been shown to alter morphology and invasiveness ^69^ in a manner similar to the epithelial to mesenchymal transition (EMT). As such, Fra-1 regulation may function as a keystone regulator, impacting several aspects of tumorigenesis and metastasis ^72^.

How MMP-1 function is coopted by tumor cells is an open question. MMP-1 is critical in degrading interstitial collagen, and tumor cells may require that function to invade ^15, 73^. However, MMP-1 has also been shown to be required for migration and xenograft tumor formation by MDA-MB-231 cells through cleavage and activation of protein activated receptor-1 (PAR-1), such that autocrine activation of specific cellular proteins is an alternative mechanism for MMP-1 function ^74^.

## Conclusion

Our work and the work of others have clearly demonstrated effects of Fra-1 and MMP-1 in multiple cancer systems. We find that in the highly metastatic MDA-MB-231 cells, Fra-1 is activated at the level of protein translation, perhaps through the loss or inhibition of an miRNA. Increased Fra-1 protein then binds the AP-1 site of the MMP-1 promoter, increasing MMP-1 transcription and translation. It is likely that other targets of Fra-1 also contribute to increased metastasis. Further research will be necessary to determine the mechanism of Fra-1 translational regulation, which target genes are involved that can lead to increased metastasis, and how these steps might be blocked to prevent metastatic progression of breast cancer.

## Figures and Tables

**Supplemental Table S1.** Top genes with greatest expression differential in genes of high metastatic potential and low metastatic potential in bone and lung. High bone metastatic cell lines are: 1833, Scp-2, Scp-25 and Scp-46. High lung metastatic cell lines are: 1834, 3481, 4142, 4173, 4175, 4180, Scp-3 and Scp-28. Low metastatic cells lines are: MDA-MB-231, Scp-6, Scp-21 and Scp-26. The gene expression values for these cell lines (Minn *et al.*, 2005) were used in a two-tailed t-test to calculate relative p-values. Genes with over three fold greater expression in bone lines and two fold in lung lines with p-values less than 0.06 are shown.

Gene	Bone (High/Low)	p-Value	Lung (High/Low)	p-Value
MMP1	98.28	0.00024	26.63	0.05619
SPANXA1/A2/B1/B2/C	18.23	0.00681	22.04	0.00570
SPANXC	14.59	0.00334	14.92	0.00756
ROBO1	13.99	0.00012	10.00	0.00013
SPANXB1B2	12.82	0.01742	15.82	0.00875
FOXA2	11.27	0.00233	9.69	0.00025
RGS2	8.32	0.00176	7.68	0.00014
NLRP3	8.24	0.00075	3.28	0.00775
CFH /// CFHR1	6.66	0.01548	5.75	0.00003
PTGS2	6.26	0.00840	5.54	0.00272
KCNK1	5.27	0.04210	3.73	0.00265
ANK3	5.05	0.02726	3.23	0.00995
KYNU	4.98	0.02145	10.12	0.01040
NR2F1	4.87	0.00088	5.36	0.00001
HAS2	4.83	0.00083	3.11	0.05186
LGR5	4.82	0.04688	3.82	0.00197
SOX4	4.67	0.03847	11.39	0.00391
RBM5	4.52	0.01639	2.13	0.05365
MOCS1	4.41	0.00053	2.36	0.00509
PRSS3	4.41	0.00751	3.21	0.00091
KHDRBS3	4.08	0.00183	2.59	0.00181
MEF2C	4.05	0.00620	2.49	0.00892
GPR37	3.99	0.02415	2.44	0.00030
ARHGDIB	3.95	0.00194	4.14	0.00001
MUSK	3.88	0.01237	3.03	0.02432
ZDHHC17	3.86	0.04522	2.49	0.05806
ODZ3	3.82	0.00299	3.61	0.00101
ENPP4	3.75	0.00026	2.88	0.00073
SULT1C2	3.74	0.00065	3.59	0.00021
MMP16	3.73	0.02076	2.15	0.04840
KCNK1	3.69	0.02844	3.01	0.00000
SLCO1B3	3.64	0.00058	2.63	0.00636
PLCE1	3.64	0.00065	2.41	0.01816
FOXA2	3.60	0.00241	3.11	0.00022
PELO	3.54	0.04780	3.28	0.02047
LPXN	3.45	0.00108	3.05	0.00005
NSBP1	3.43	0.05792	2.95	0.01074
CASP1	3.34	0.01960	2.32	0.04582
PRSS3	3.34	0.02957	2.43	0.00078
TSPAN13	3.28	0.01774	2.55	0.00022
MAGEH1	3.25	0.04480	2.03	0.03014
ANGPTL4	3.19	0.04369	3.68	0.00043
NR3C2	3.12	0.00236	2.18	0.01802
FLJ20489	3.07	0.02672	2.76	0.05765
SERPINI1	3.07	0.02671	2.87	0.00013
