# Supplementary material for: Fra-1 regulation of Matrix Metallopeptidase-1 (MMP-1) in metastatic variants of MDA-MB-231 breast cancer cells
Source: F1000Res. 2013 Oct 29;2:229. [Version 1] doi: 10.12688/f1000research.2-229.v1 (PMC4193399; doi:10.12688/f1000research.2-229.v1)
Supplement: Peer review history

# Peer review: Referee response for version 1

**DOI:** 10.5256/f1000research.2686.r4351

**Published:** 2014-04-22

**Authors:** Danny N Dhanasekaran

**Affiliations:** 1 OU Cancer Institute, University of Oklahoma Health Sciences Center, Oklahoma City, OK, USA

This is a well-designed study with solid data to support the claim that Fra-1 regulated MMP-1 is involved in the metastatic phenotype of a variant cell line derived from MDA-MB-231 cells.  The study is carried out with well-controlled experiments.  The authors have nicely presented their voluminous data with a very succinct discussion.

I have read this submission. I believe that I have an appropriate level of expertise to confirm that it is of an acceptable scientific standard.

# Peer review: Referee response for version 1

**DOI:** 10.5256/f1000research.2686.r2520

**Published:** 2013-11-26

**Authors:** Sekhar Reddy

**Affiliations:** 1 University of Illinois Cancer Center, University of Illinois at Chicago, Chicago, IL, USA

Increased expression levels of matrix metallopeptidase 1 (MMP-1) and the FRA-1/AP-1 transcription factor have been implicated in breast cancer cell progression. This manuscript by Henckels and Prywes addresses the mechanisms underlying the transcriptional activation of MMP-1 by FRA-1 in metastatic variants of breast adenocarcinoma cells. They have performed comprehensive experiments to delineate the upregulation of MMP-1 in breast cancer cells and found that FRA-1 is required for the transcriptional induction of MMP-1 and metastasis of the highly invasive cancer cell line, Scp-2. While these studies confirm previous findings that FRA-1 regulates MMP-1 expression and breast cancer cell metastasis and that FRA-1 mRNA expression is greater in breast cancer cells; the discovery that FRA-1 translation is an additional regulatory mechanism that contributes to the increase in MMP-1 expression in breast cancer cells is novel and significant. They have performed elegant studies and demonstrated that neither the mRNA expression and degradation nor protein stability of FRA-1 are altered, but its protein synthesis rate is increased, leading to increased FRA-1 expression in these highly metastatic cells. Furthermore, they have performed depletion studies with RNAi to address the role of Jun family members and have shown that c-Jun (but not Jun-B or Jun-D) is a potential dimeric partner of FRA-1 in the regulation of MMP-1 expression. While their studies clearly support the authors’ overall conclusions, additional experiments involving ChIP and re-ChIP assays with c-Jun antibody could have further strengthened the EMSA results and indicated whether the “FRA-1/c-Jun” or “FRA-1/X” complex regulates the MMP-1 transcription. It is also unclear whether depletion of MMP-1 phenocopies the effects of FRA-1-siRNA knockdown in Scp-2 cells. Nonetheless, the study raises an important issue: that increased FRA-1 expression is differentially regulated in breast cancer cells. Whether such (transcriptional and translational) regulation of FRA-1 is indicative of different grades of breast metastasis is a question worth pursuing. Further defining the mechanisms that underlie increased FRA-1 expression levels is critical to devising novel strategies for breast cancer treatment.

I have read this submission. I believe that I have an appropriate level of expertise to confirm that it is of an acceptable scientific standard.
